# Effects of cognitive training based on metamemory and mental images

**DOI:** 10.1590/S1980-57642010DN40200007

**Published:** 2010

**Authors:** Thaís Bento Lima-Silva, Tiago Nascimento Ordonez, Glenda Dias dos Santos, Aline Teixeira Fabrício, Flávia Ogava Aramaki, Evany Bettine de Almeida, Débora Lee Vianna-Paulo, Mayne Patrício Malagutti, Ana Carolina Valente-Oliveira, Amanda Iwasaki, Gisele dos Santos Souza, Mônica Sanches Yassuda

**Affiliations:** 1 Bachelors in Gerontology, School of Arts, Sciences and Humanities, University of São Paulo, São Paulo SP, Brazil.; 2 Undergraduate Students in Gerontology, School of Arts, Sciences and Humanities, University of São Paulo, São Paulo SP, Brazil.; 3 Assistant Professor in Gerontology, School of Arts, Sciences and Humanities, University of São Paulo, São Paulo SP, Brazil.

**Keywords:** cognitive training, cognition, mental images, self-efficacy, metamemory, elderly, aging

## Abstract

There is scant research evidence regarding training effects among elderly with limited educational experience. Research indicating an association between metamemory and memory performance is based on samples of older adults with at least 12 years of education.

**Objectives:**

To test the efficacy of a cognitive training program based on the creation of mental images and changes in specific aspects of metamemory in individuals with 3 to 15 years of education (M=8.38, SD=4.24).

**Methods:**

37 older adults participated in five training sessions (Training Group (TG)) and 32 control subjects completed only pre and post test assessments (Control Group (CG)) including the Mini Mental Status Examination (MMSE), the Geriatric Depression Scale (GDS), the Brief Cognitive Screening Battery (BCSB)(naming and memorization of 10 pictures, animal category verbal fluency test, the Clock Drawing Test (CDT)), the Story subtest from the Rivermead Behavioural Memory Test (RBMT), the Memory Complaint Questionnaire (MAC-Q), and the Picture and Story domains from the Memory Self-Efficacy Questionnaire (MSEQ).

**Results:**

The TG showed significant improvement between pre and post tests on the delayed recall of the 10 pictures and in self-efficacy for the memorization of stories. These same changes were not found in the CG.

**Conclusions:**

Five-session cognitive training may lead to significant improvements in episodic memory and memory self-efficacy, an aspect of metamemory, in individuals with an average of 8 years of education.

## Abstract (pt)

Existem na literatura poucas evidências sobre os efeitos do treino cognitivo entre idosos com baixa escolaridade. Pesquisas que evidenciam a associação entre metamemória e o desempenho em tarefas de memória baseiam-se em amostras com idosos com 12 anos de escolaridade ou mais

**Objetivos:**

Testar a eficácia de um programa de treino cognitivo baseado na criação de imagens mentais e na alteração de aspectos da metamemória entre indivíduos entre 3 e 15 anos de escolaridade (M=8.38, SD=4.24).

**Métodos:**

37 adultos maduros e idosos participaram de cinco sessões de treino (Grupo Treino (GT)) e 32 somente completaram as avaliações pré e pós-intervenção (Grupo Controle (GC)), com o Mini Exame do Estado Mental (MEEM), a Escala de Depressão Geriátrica (EDG), a Bateria Breve de Rastreio Cognitivo (BBRC) (nomeação e memorização de 10 figuras, fluência verbal animais, Teste do Desenho do Relógio), o subteste Estória do Teste Comportamental de Memória de Rivermead (RBMT), o Questionário de Queixas de Memória (MAC-Q), os domínios Figura e História do Questionário de Auto-Eficácia para Memória (MSEQ).

**Resultados:**

O GT apresentou ganho significativo entre o pré e o pós-teste no resgate tardio das 10 figuras e na auto-eficácia para a memorização de histórias. Estas alterações não foram encontradas no GC.

**Conclusões:**

O treino cognitivo de cinco sessões pode gerar melhora significativa em memória episódica e em auto-eficácia, um aspecto da metamemória, em indivíduos com 8 anos de escolaridade em média.

Studies on cognitive plasticity have proven a fertile field for basic research since they yield essential information about human aging. Moreover, these studies have important implications for the clinical practice of gerontologists because optimizing memory is associated to health, autonomy and independence in the elderly.^1^

Studies investigating cognitive training differ not only in terms of duration, but specifically on the strategies taught and training methodologies employed. Thus, the results concerning the magnitude of effects, generalization for untrained tasks and long-term retention reported in the literature vary widely.^2^ Nevertheless, there is consensus that cognitive interventions can lead to significant performance improvements^3^ and recent data suggests that cognitive training can reduce age-associated functional decline.^4^

There is scant data from Brazilian studies regarding the efficacy of cognitive interventions in our elderly population, which tends to have lower levels of formal education. However, a recent study has reported significant gains in memory performance in older adults with four to eight years of schooling who underwent five training sessions involving categorization strategies.^5^ Participants who practiced using categorization with pictures and grocery lists outperformed control participants in similar tasks at post test. In another study involving illiterate and semi-literate older adults who received eight sessions of training based on categorization or mental images, results indicated that mental images might be more effective to improve memory performance among elderly with limited education.^6^

Memorization strategies based on the creation of interactive mental images have been described in previous successful studies on memory rehabilitation and training.^7-9^ These strategies stimulate the creation of a link between mental images and the information to be memorized. Although creating mental images may require cognitive resources such as concentration, they have been proven to be highly effective among older adults.^7-9^

Cognitive training studies also highlight that cognitive performance can be modulated by variables related to metamemory.^10^ The term metamemory originally referred to a broad array of knowledge held about the workings of memory.^11^ More recently, metamemory has been described as a latent construct which includes knowledge, perceptions and beliefs about one’s own memory and about memory systems in general.^12^ In aging studies, a particular dimension of metamemory that has been regarded as relevant for daily living is memory self-efficacy.^12,13^ Memory self-efficacy refers to the level of confidence that an individual has in his/her own abilities to perform memory tasks^10^. In previous aging studies it has been related with motivation to perform memory tasks, degree of effort invested, use of strategies and perseverance on tasks.^10-13^ A recent study has also shown that memory self-efficacy at baseline could predict memory performance levels after six years.^14^

Cognitive interventions have targeted metamemory, including knowledge about memory and memory self-efficacy, as a means of enhancing the impact of training.^15^ Several training studies have attempted to educate older adults about memory and aging, alter negative beliefs about memory performance in later life, and improve memory self-efficacy, since metamemory has been associated with memory performance (for a review of this literature, refer to Dunlosky and Hertzog,^9^ in Brazil, refer to Yassuda et al.^16^).

There is limited research evidence regarding training effects among elderly with limited educational experience. Research indicating the association between metamemory and memory performance is based on samples of older adults with at least 12 years of education. Therefore, the aim of this study was to test the efficacy of a cognitive training program based on the creation of mental images and on the alteration of certain metamemory aspects, that is, perception of change and memory self-efficacy. This study sought to investigate whether an intervention containing these two elements could generate simultaneous or synergetic effects on cognitive performance and on metamemory measures. More specifically, the aim was to test whether this combined approach could generate significant effects among older adults with limited education.

## Methods

### Participants

A total of 69 older adults with 3 to 15 years of schooling (M=8.38, SD=4.24) and enrolled in a university program for seniors took part in the study. Participants were randomly subdivided into a Training Group (TG) and a Control Group (CG). The TG comprised 37 subjects who received five training sessions, whereas the CG comprised 32 participants who did not participate in any training sessions. Subjects from both groups were submitted to pre and post-intervention assessments. Participants from the CG were later enrolled in a forthcoming intervention. There was no placebo condition to control for social interaction effects, and this is a limitation of the study.

### Materials

All subjects underwent an initial assessment involving signing the Informed Consent form, completion of a socio-demographic questionnaire, and administration of the following instruments: Mini Mental State Exam (MMSE),^17^ Geriatric Depression Scale with 15 questions (GDS),^18^ the Brief Cognitive Screening Battery (BCSB)^19^ (naming and memorization of 10 pictures, animal category verbal fluency test, the Clock Drawing Test (CDT)), the Story subtest from the Rivermead Behavioural Memory Test (RBMT),^20^ the Memory Complaint Questionnaire (MAC-Q),^21,22^ and the Picture and Story domains from the Memory Self-Efficacy Questionnaire (MSEQ).^16^

MMSE scores range from 0 to 30 points and the following cut-off scores were adopted according to schooling level: for illiterates, 17 points; individuals with 1 to 4 years of schooling, 20 points; 5 to 8 years, 24 points; greater than 8 years of education, 26 points. These cut-off scores were adapted from Brucki et al.^17^ by calculating a standard deviation from the mean for each schooling level, and then rounding them down. In the present study, a cut-off score of 6 was adopted for the version of the GDS, with scores ranging from 0 to 15 points. The elderly individuals included in the sample presented scores which indicated absence of dementia (MMSE and BCSB) and no severe depression (GDS>10). Only one older adult presented a score of 12 on the GDS during the pre-test. Data for this participant was maintained in study analyses because there was cognitive preservation in the MMSE and in the BCSB.

For the BCSB, performance was assessed based on the number of pictures named correctly (0-10), the number of pictures correctly recalled in incidental memory (after naming, unaware of the memory task) (0-10), the number of pictures recalled after studying them for 30-seconds-immediate memory (0-10), and the number of pictures correctly recalled (0-10) after performing the verbal fluency task, and the Clock Drawing Test - delayed recall. We decided to omit the third chance to memorize the pictures (Learning) in order to reduce the risk of reaching ceiling effects at post-test. Verbal fluency consisted of counting the names of the animals mentioned, according to criteria by Brucki and Rocha.^23^ The CDT was corrected based on the criteria by Sunderland and colleagues,^24^ with scores between 0 and 10.

The MAC-Q scale comprises six questions which aim to assess the perception of decline in memory tasks (in ten years for this study) involving five everyday situations, such as remembering telephone numbers or codes used on a daily or weekly basis, and a final question on overall mnemonic performance. Scores ranged from 0 to 35 points with higher scores being suggestive of greater concern about memory decline.

The MSEQ scale assesses the degree of confidence a person presents when performing certain memory tasks. The Picture and Story domains were selected because they were the tasks that matched most closely to the memory tasks contained in the protocol. At increasing levels of difficulty, individuals indicate if they would be able to complete a memorization task and if so, the degree of confidence with which they believe they could carry out the task. The scale produces two variables: the total number of times the person states they are able to perform the task (0-5 for each domain) and percentage means indicating the degree of confidence in the ability to carry out the tasks (0-100% for each domain). Only percentage means were analyzed in this study.

### Procedures

Each of the five training sessions included a 45-minute educational intervention aimed at offering information about memory and aging and at changing negative beliefs. This educational component followed a pre-determined protocol. In each session different memory sub-systems were explored, and aspects of memory which tend to remain stable with aging were highlighted. Special attention was given to the stability observed in immediate memory, semantic memory, and implicit memory. Everyday examples of tasks involving these sub-systems were used in order to avoid technical terms. In all sessions, participants were asked to report memory successes, i.e. occasions on which they managed to remember important information, with the objective of improving self-efficacy. After the educational intervention, participants were divided into small groups, led by Gerontology students, and spent 45 minutes learning to use mental images for memorizing words, phrases and short stories. Initially, they learned to use mental images to memorize single words, and short phrases. In the last two sessions they practiced using mental images to remember short stories. In all sessions, between the study phase and the recall phase, participants were challenged to perform fluency tasks, with categorical and phonemic restrictions. These tasks were carried out in a group format, and participants were enthusiastic about them. The students also followed a pre-determined protocol, and received training to reduce discrepancies among the groups.

### Statistical analyses

Frequency tables for categorical variables (gender) provide the profile of the sample. Descriptive statistics (mean, standard deviation, minimum and maximum values) for the continuous variables were calculated, including scores for cognitive and self-efficacy scales. The chi-square test was employed in order to compare the categorical variable gender among groups. Student’s t test for independent samples was used to compare continuous variables between the two groups, when these were normally distributed. In order to assess changes from pre to post-test scores, deltas were calculated (post-test score subtracted from pre-test score) and the deltas for both groups were then compared using Student’s t test for independent samples. To confirm these results, repeated measures ANOVAs were also carried out, with Time as a within-subject factor (pre x post test) and Group as between-subject factor (TG x CG), with Tukey post hoc testing. The level of significance adopted for the statistical tests was 5% (p<0.05). Version 9.0 of the SPSS statistical package was used for all statistical analyses.

## Results

Table 1 below presents the socio-demographic data for the total sample. With the exception of gender (there was a higher proportion of men in the CG), no significant difference was detected between the groups, i.e. the social demographic profiles of both groups were found to be similar for age, education and income. However, a significant difference was identified for MMSE and GDS - participants from the TG had higher performance on the MMSE and less depressive symptoms. The sample did not include illiterates, since all participants had at least three years of formal education.

**Table 1:** Means and standard deviation for sociodemographic data.

	TG (37)	CG (32)	p-value
Age	65.32 (6.11)	63.66 (5.78)	0.251^a^
Schooling	8.95 (4.04)	7.97 (4.27)	0.333^a^
Income	2.92 (1.74)	2.41 (1.24)	0.169^a^
MMSE	27.35 (2.34)	25.75 (2.60)	0.009^a^
GDS	2.68 (1.97)	3.88 (2.64)	0.035^a^
No. of women	34	22	0.032^b^

a p-value refers to the Student t test for independent samples;

b p-value refers to the chi-square test.

The comparison of deltas of the TG and CG revealed a significant difference for MSEQ-Story and Delayed Memory on the BCSB. These data are shown in Table 2.

**Table 2:** Means and standard deviation (in parenthesis) for dependent variables for the TG (n=37) and the CG (n=32).

Group	Pre-test (n=69)	Post-test (n=69)	Delta
MSEQ Pictures			
Training	46.00 (16.08)	50.70 (18.07)	4.70 (18.61)
Control	42.75 (19.81)	44.06 (21.02)	1.31 (18.40)
MSEQ Story			
Training	54.81 (21.70)	63.24 (21.37)	8.43 (24.20)^a^
Control	59.62 (37.07)	50.70 (23.81)	-8.93 (26.00)^a^
MAC-Q			
Training	25.11 (4.03)	23.76 (4.30)	-1.35 (5.36)
Control	24.66 (6.13)	25.13 (0.20)	0.46 (3.29)
BCSB Naming			
Training	9.97 (0.16)	10.00 (0.00)	0.03 (0.16)
Control	9.97 (0.18)	9.97 (0.18)	0.00 (0.25)
BCSB Incidental Mem			
Training	6.16 (1.19)	7.54 (1.22)	1.38 (1.60)
Control	6.19 (1.60)	7.09 (1.28)	0.90 (1.78)
BCSB Immediate Mem			
Training	8.65 (1.18)	9.14 (1.06)	0.49 (1.04)
Control	8.50 (1.44)	8.72 (1.25)	0.22 (1.16)
BCSB Delayed Mem			
Training	7.78 (1.46)	9.03 (0.96)	1.25 (1.46)^a^
Control	7.69 (1.67)	7.91 (1.71)	0.21 (1.84)^a^
Verbal Fluency			
Training	15.51 (3.19)	16.70 (3.27)	1.89 (3.52)
Control	14.00 (4.50)	14.63 (4.43)	0.62 (3.50)
Clock Drawing Test			
Training	8.32 (1.40)	8.49 (1.41)	0.16 (1.59)
Control	6.41 (3.15)	7.22 (2.49)	0.81 (0.72)
Story RBMT			
Training	6.54 (1.54)	7.14 (2.71)	0.60 (2.82)
Control	5.25 (2.18)	5.22 (2.04)	-0.03(2.25)
Delayed Recall Story RBMT			
Training	5.86 (1.84)	6.78 (2.50)	0.92 (2.70)
Control	4.78 (2.22)	4.81 (2.38)	0.04 (2.96)
GDS			
Training	2.68 (1.97)	2.57 (1.86)	-0.19 (1.62)
Control	3.88 (2.64)	3.31 (2.31)	-0.56 (1.31)

a Indicates statistically significant difference between groups for deltas on Student's t test for independent samples, p<0.05.

Repeated measures ANOVAS indicated a significant Time x Group interaction. Post hoc testing suggested that only the TG demonstrated performance increments from pre to post test, for MSEQ-Story and Delayed Memory on the BCSB. Results from ANOVAs and the delta analyses were congruent (Table 3).

**Table 3:** Results for the ANOVAS comparing scores for the TG and the CG at pre and post test.

Variables	Group (TG × CG)	Time (Pre × Post Test)	Interaction effects (Group × Time)
MSEQ Pictures	F_(1,67)_=1.59; p=0.212	F_(1,67)_=1.81; p=0.183	F_(1,67)_=0.58; p=0.451
MSEQ Story	F_(1,67)_=0.56; p=0.458	F_(1,67)_=0.01; p=0.934	F_(1,67)_=8.25; p=0.005^a^
MAC-Q	F_(1,67)_=0.17; p=0.686	F_(1,67)_=0.65; p=0.422	F_(1,67)_=2.78; p=0.100
BCSB Naming	F_(1,67)_=0.51; p=0.478	F_(1,67)_=0.28; p=0.597	F_(1,67)_=0.28; p=0.597
BCSB Incidental Mem	F_(1,67)_=0.74; p=0.393	F_(1,67)_=31.35; p=<0.001^b^	F_(1,67)_=1.34; p=0.251
BCSB Immediate Mem	F_(1,67)_=1.13; p=0.292	F_(1,67)_=7.08; p=0.010^c^	F_(1,67)_=1.02; p=0.316
BCSB Delayed Mem	F_(1,67)_=4.35; p=0.041^d^	F_(1,67)_=13.48; p=<0.001^d^	F_(1,67)_=6.62; p=0.012^d^
Verbal Fluency	F_(1,67)_=4.71; p=0.034^e^	F_(1,67)_=4.59; p=0.036^e^	F_(1,67)_=0.44; p=0.508
Clock Drawing Test	F_(1,67)_=12.14; p=0.001^f^	F_(1,67)_=3.41; p=0.069	F_(1,67)_=1.52; p=0.222
Story RBMT	F_(1,67)_=14.60; p=<0.001^g^	F_(1,67)_=0.82; p=0.368	F_(1,67)_=1.01; p=0.317
Delayed Recall Story RBMT	F_(1,67)_=13.15; p=0.001^h^	F_(1,67)_=1.94; p=0.168	F_(1,67)_=1.70; p=0.197
GDS	F_(1,67)_=3.81; p=0.055	F_(1,67)_=3.46; p=0.067	F_(1,67)_=1.59; p=0.212
MMSE	F_(1,67)_=9.34; p=0.003^i^	F_(1,67)_=5.54; p=0.022^i^	F_(1,67)_=0.02; p=0.903

Note: F(a,b)= F statistics with "a" as degrees of freedom in the numerator and "b" as degrees of freedom in the denominador. N=61.

a Significant interaction, Tukey test TG: Pre<Post (p=0.041); CG: Pre=Post (p=0.061);

b Significant differences for both groups: Pre<Post (TG: p<0.001 and CG: p=0.007);

c TG: Pre<Post (p=0.007); CG: Pre=Post (p=0.293);

d Significant differences, TG: Pre<Post (p<0.001); CG: Pre=Post (p=0.507);

e Significant differences, TG: Pre<Post (p=0.048); CG: Pre=Post (p=0.319);

f Significant differences, TG>CG, at pre (p>0.001) and post (p=0.010);

g Significant differences, TG>CG, pre (p>0.006) and post (p=0.002);

h Significant differences, TG: Post>Pre (p=0.046); CG: Pre=Post (p=0.953);

i Significant differences, TG: Post=Pre (p=0.146); CG: Pre=Post (p=0.060).

## Discussion

The aim of this study was to test the efficacy of a cognitive training program based on the creation of mental images and the alteration of meta-memory, particularly perception of cognitive chance and memory self-efficacy. The results suggest that the educational intervention concerning aspects of memory which tend not to change during aging, and highlighting of successful situations involving memory, can bring about positive change in memory self-efficacy. Furthermore, the results suggest that mental image-based training can lead to benefits in tasks demanding episodic memory.

The use of mental images in memory training studies in aging are now commonplace. Studies by Yesavage and colleagues^8^ conducted in the 1980s demonstrated the efficacy of mental images for memorizing face and name pairs. However, compared to the use of verbal mediators, mental images are relatively less studied as memory aids.^25^ There is very limited information on their efficacy when used by older adults with less than 12 years of education.

In a training study based on the creation of mental images and categorization, Salmazo and Yassuda^6^ demonstrated that the use of mental images can be effective for improving performance on episodic memory tasks among elderly who are illiterate or have very low education. Therefore, the present study contributes to the current training literature, suggesting that mental images may also lead to performance changes among elders with an average of 8 years of education.

The method of creating mental images employed in the present study differed from methods used in previous studies. Yesavage^26^ used images by famous painters and highlighted the differences and similarities between the works of art. In the study by Brooks III et al.,^27^ creating mental images was a stage preceding the teaching of two types of strategies, namely, face-name associations and the method of loci. The elderly were asked to analyze the images and associate short verbal sentences intended to facilitate name recall in the face-name strategy or the order of items in the method of loci strategy. The results revealed that, although the visualization pre-training was given prior to the strategies training for both groups, the group which trained using the method of loci showed greater improvement in the post-test than the group which practiced with the face-name strategy. In the cited study, after teaching both memorization strategies, a memory questionnaire was applied before and after the interventions. The authors found that participants had a greater knowledge on strategies after the training. By contrast, in the present study, knowledge about memory was not assessed and this represents a study limitation since participants might have shown changes in this important aspect of metamemory due to the educational component of the intervention.

Previous studies have indicated that training programs which incorporate principles of self-efficacy theory have a significant potential to improve older adults’ cognitive abilities.^15^ In the present study, some aspects of the intervention were in line with self-efficacy theory, such as the fact that aspects of memory which do not change significantly in later life were discussed in every session, and that successful memory experiences were constantly brought to the attention of the class. In addition, memory exercises were organized in such a way that easier tasks always preceded difficult ones, enhancing older adults’ confidence in their memory abilities. Current findings support the results from West and colleagues,^15^ as they also suggest that integrated training programs which target mnemonic techniques as well as aspects of metamemory may be beneficial for seniors who frequently worry about their memory performance.

In the present study, older adults in the TG were encouraged to create mental models in episodic memory exercises. Subjects were exposed to words, short phrases and stories, and were encouraged to highlight the differences and similarities between the imagery created by each participant. Participants may have used the creation of mental images in the post-test and this in turn could explain the enhanced performance in the delayed recall of pictures. However, the use of strategies was not objectively assessed in the present study and constitutes another study limitation. Future training studies should include strategy use measures. Other limitations include the relatively low number of training sessions, since more sessions might have led to greater improvements. Further training studies should be conducted involving a higher number of sessions and longitudinal follow up assessments to check if training gains persist over time. Nonetheless, this study can contribute to the gerontological literature in that it involved community dwelling older adults, used scales previously adapted for the Brazilian elderly population, and included a control group. Preserved cognitive function can improve the quality of life among the elderly because it allows them to remain active and independent longer.^4^ Therefore, further studies are warranted on this increasingly important topic.
